# Age-related differences in eating location, food source location, and timing of snack intake among U.S. children 1–19 years

**DOI:** 10.1186/s12966-023-01489-z

**Published:** 2023-07-26

**Authors:** Gina L. Tripicchio, Christina M. Croce, Donna L. Coffman, Cameron Pettinato, Jennifer O. Fisher

**Affiliations:** 1 Department of Social and Behavioral Sciences, Center for Obesity Research and Education, College of Public Health, Temple University, 3223 N. Broad Street, Suite 175, Philadelphia, PA 19140 USA; 2 Department of Psychology, College of Arts and Sciences, University of South Carolina, Columbia, SC USA

**Keywords:** Snacking, Eating behavior, Food source location, Time of day, Children, Toddlers, Preschoolers, Adolescents

## Abstract

**Background:**

Snacking is nearly universal and contributes significant energy to U.S. children’s diets. Little is known, however, about where and when snacking intake occurs and if such patterns change with age. This research evaluated age-related differences in eating location, food source location, and timing of snacking among U.S. children aged 1–19 years (y).

**Methods:**

A cross-sectional analysis of snacking among 14,666 children in the 2007–2018 U.S. National Health and Nutrition Examination Survey was conducted using a single 24-hour dietary recall. Snacking was participant-defined and included all eating occasions outside of meals. Linear regression and analysis of covariance were used to examine the effects of age (toddler 1–2 y, preschooler 3–5 y, school-age 6–11 y, adolescent 12–19 y) on the percentage of daily snack energy consumed by eating location (at home vs. away from home), food source location (grocery store, convenience store, school/childcare, restaurant, from someone else (i.e. “socially”), and other), and time of day (morning, 6am-12pm; early afternoon, 12pm-3pm; late afternoon/afterschool 3pm-6pm; evening 6pm-9pm, late-night 9pm-12am, and overnight 12am-6am).

**Results:**

On average, U.S. children consumed most of their daily snacking energy at home (71%), from foods and beverages obtained from grocery stores (75%), and in the late afternoon/afterschool (31%). Toddlers and preschoolers consumed a greater percentage of their daily snack energy during the morning hours compared to school-age children and adolescents (both p < 0.001); school-age children consumed the most in the evening (27%, p < 0.001), and adolescents consumed the most in the late-night period (22%, p < 0.001). Age-related increases were seen in the percentage of daily snacking energy eaten outside the home (p < 0.001), and obtained socially (p < 0.001), from restaurants (p < 0.001), and convenience stores (p < 0.001).

**Conclusion:**

Findings reveal age-related differences in eating location, food source location, and timing of snack intake among U.S. children aged 1–19 y. Younger children consume a greater percentage of snacking calories in the morning and at home relative to older children. School-age children and adolescents show greater snacking in the evening and at night and from foods obtained and eaten outside the home. Efforts to promote healthy snacking behaviors among children should consider developmental differences in snacking patterns.

**Supplementary Information:**

The online version contains supplementary material available at 10.1186/s12966-023-01489-z.

## Background

An overwhelming majority of children in the United States (U.S.) consume snacks (i.e., foods and beverages outside of structured meals), contributing significant energy to children’s diets [1, 2]. Most recent data from the 2017–2020 U.S. National Health and Nutrition Examination Survey (NHANES) demonstrate that 93% of children 2–19 years (y), consume at least one snack per day and snacks provide one-quarter (25%) of total daily energy [3]. Snacking is also common among children internationally; the prevalence of snacking in Australia and Canada mirrors that of the U.S with more than 95% of children consuming snacks daily [2]. Moreover, even in countries where snacking rates are lower like China, Mexico, and Brazil, the prevalence of snacking has significantly increased in recent years, highlighting the public health relevance of this behavior [4–7]. Healthy snacks are endorsed by leading professional organizations and government entities to help children meet nutrient requirements for growth; however, snacking is also a significant source of fat and added sugar in children’s diets, leading to concerns about the role of snacking in excessive intake and obesity [2, 8]. Worldwide, there is a call for consensus around definitions of snacking and clearer recommendations on how to include snacks as part of a healthful diet [9].

It is well established that nutrient needs, eating behaviors, and social contexts around eating change significantly across the course of childhood [10]. Snacking appears to have a significant developmental component as well. Snacking among young children, for instance, is endorsed by leading professional organizations, reflecting the assumption that young children need snacks to meet nutrient needs [11]. This assumption underlies federal funding of healthy snacking in childcare and early childhood educational settings in the U.S., such as the Child and Adult Care Food Program (CACFP) [12]. In contrast, the necessity of snacks among older school-aged children and adolescents has been debated, particularly the quality of foods and beverages purchased at school and stores outside of school [13]. For instance, among 4-6th grade children, surveys of children’s purchases at corner stores revealed that energy-dense, low-nutritive foods and beverages were the most frequently purchased items [14]. Additionally, media and peer influences on snacking may also be pronounced among older children. For instance, individual intakes of snacks and sugar-sweetened beverages (SSBs) among adolescents were positively associated with intakes of their peer groups at school [15]. While these studies point to contextual influences on snacking behavior, there have been few systematic efforts to understand where and when snacking intake occurs among children and how these patterns may change with age. Characterizing contextual influences on snacking by developmental stage provides important data to inform anticipatory guidance as well as interventions targeting snacking to improve diet quality and reduce obesity risk in children.

Previous research has found that the majority of snacking intake among U.S. children occurs at home, but it remains unclear if patterns of snacking differ across the day or vary by age group [16, 17]. Additionally, grocery stores have been previously identified as the primary food location source (i.e., location from which foods are obtained) of overconsumed nutrients in children’s diets [18–20]. However, this work has not specifically examined snacking. Similarly, developmental influences on the timing of snack intake among U.S. children are not well elucidated. A nationally representative study of U.S. children 4–13 y of age found that the afternoon was the most common time when snacks were consumed, followed by the evening, and the morning period was when the fewest snack calories were consumed [21]. However, age related differences in timing were not considered.

To date, our team has conducted a number of analyses of NHANES data using comprehensive definitions of snacking (i.e., snack frequency (number of snacks per day), snack size (kcal/snack), and snack energy density (kcal/g/snack)) and examined associations with weight status and diet quality. We have conducted analyses in nationally representative U.S. samples of young children (1–5 years) and adolescents (12–19 years) and found that in both age groups children with overweight and obesity consume more snacks per day and more calories per snack than children with normal weight, and that snacking contributes significantly to children’s intake of added sugar, saturated fat, and sodium [22–25]. Moreover, in adolescents, we have identified that the top food group contributions from snack intake are added sugars, solid fats, refined grains, and fruits[26]. While evidence to date indicates that snacking is a key dietary behavior that impacts energy balance and diet quality in childhood, little is known about the context in which children consume snacks and how this might change with development. Thus, to address the current gaps in understanding of developmental and contextual influences on snacking, this research evaluated age-related differences in eating location, food source location, and timing of snacking among U.S. children aged 1–19 y. Specifically, the percentage of daily energy from snacking occasions was evaluated for toddlers (1–2 y), preschoolers (3–5 y), school-aged children (6–11 y), and adolescents (12–19 y) by eating location, food source location, and time of day using nationally representative NHANES data from the most recent decade.

## Methods

### Study design and participants

A secondary, cross-sectional analysis of NHANES data from children 1–19 y, collected over the most recent decade (six combined cycles; 2007–2018) was conducted. NHANES is an ongoing, nationally representative study of the nutritional and health status of the civilian, non-institutionalized U.S. population [27]. NHANES uses a complex, multistage, probability sampling design with county as the primary sampling unit from which clusters of households and participants are randomly selected [28, 29]. Written informed consent is obtained from parents for all children 2–17 y, assent is obtained for children 7–17 y, and written informed consent is obtained for children 18–19 y. NHANES assesses nutritional status using dietary recalls, anthropometric measurements, laboratory tests, and clinical examinations. The present analysis used a single 24-hour dietary recall to assess snack energy intakes, locations, and time of day. Survey weights adjusting for dietary-interview-specific non-response and the day of the week were applied. Other design and data collection procedures for NHANES are detailed elsewhere [28, 30]. The National Center for Health Statistics Research Ethics Review Board approved the study protocols.

### Measures

#### Snacking energy

Following NHANES analytic guidance for estimating group mean intakes, dietary intake was assessed using a single 24-hour dietary recall collected by trained interviewers using the U.S. Department of Agriculture What We Eat in America Survey’s 5-step Automated Multiple-Pass Method [30, 31]. The 24-hour recall was collected in person at the NHANES Mobile Examination Center [28]. Dietary recall assessment methodology varied by the age of the child: for young children ≤ 5 y, parents or a proxy familiar with the child’s intake provided the dietary recalls; for children 6–11 y, recalls were proxy assisted with parents; children 12–19 y completed recalls on their own.

Snacking occasions were participant-defined from a pre-determined list that included the following categories: “snacks,” “beverages” not otherwise included in meals, or “extended consumption,” as well as the Spanish equivalents of “*merienda*”, “*bebida*”, “*botana*”, “*bocadillo*”, “*tentempie*”, and “*entre comida.*” These categories were used to capture all eating and drinking that occurred outside of meals. Individual snack food and beverage items were retained and separately marked by eating location, food source location, and time of consumption; foods consumed at the same time could be sourced from different locations. The percentage of daily snack energy for each eating location, food source location, and time of day category was calculated by taking the sum of snack energy for each category (kcal) and dividing by total daily snack energy (kcal).

#### Snacking location and timing

Eating location was used to indicate whether each food and beverage was consumed at home or away from home. Food source location indicated where each food and beverage item was obtained (e.g., grocery store) and was categorized by the interviewer using a predefined list (Supplementary File 1). From the initial 30 NHANES response options, food source locations were divided into 6 categories: (1) Grocery stores; (2) Convenience stores; (3) School and childcare centers; (4) Restaurants including fast food and full-service; (5) Social, which included foods and beverages obtained “from someone else/gift”; and (6) Other, representing all other options. Time of day categories were derived from previous studies: morning (6am-12pm); early afternoon, (12pm − 3pm); late afternoon/afterschool (3pm–6pm); evening (6pm–9pm), late-night (9pm-12am), and overnight (12am-6am) [32, 33]. Late afternoon/afterschool and late-night categories were distinguished to reflect previous research showing associations between eating within those windows to consumption of energy-dense nutrient poor foods and obesity risk, respectively among children [8, 34, 35].

#### Socio-demographics

Socio-demographic characteristics, collected by self-report using the NHANES computer-assisted personal interviewing system, were included as covariates in this analysis: child age (y), child gender (male, female), child race and ethnicity (Hispanic/Mexican American, non-Hispanic Black, non-Hispanic White, other/multi-racial/non-Hispanic Asian), and head of household (HH) age (< 40 y and ≥ 40 y), HH education (less than high school, high school/some college, college graduate or above), HH marital status (partnered vs. not-partnered), and ratio of poverty-to-income (PIR; <125% vs. > 125% of the poverty guidelines, representing above and below the poverty thresholds for determining eligibility to receive government assistance) [36]. Race and ethnicity categories were grouped as noted above; non-Hispanic Asian is included in the “other” category because this analysis includes data from two survey cycles prior to 2011 when NHANES began to provide separate estimates for this group [30]. Age categories were determined based on NHANES recommendations to reduce variability in the sample weights (5 and under, 6–11 y, 12–19 y) [37]. The category “5 y and under” was further split into 1–2 y and 3–5 y to distinguish the developmentally and nutritionally unique period of toddlerhood (e.g., 1–2 y) from early childhood (e.g., 3–5 y) [10].

### Statistical analysis

Analyses were performed in R version 4.2.1 (RStudio, Inc.) [38]. Per NHANES analytical guidelines, sample weights were created by dividing the day one dietary sample weights by six for each of the six two-year cycles of NHANES data combined in this analysis [39, 40]. Descriptive statistics were generated for all variables of interest. Generalized linear regression models were used to evaluate the percentage of daily snack energy by age-group separately for each category: eating location (at home vs. away from home), food source location (grocery store, convenience store, school/childcare, restaurant, social, other), and time of day (morning, early afternoon, late afternoon/afterschool, evening, late-night, overnight). Each model was adjusted for survey cycle, and demographic characteristics. Child gender, child race and ethnicity, HH age, HH education, HH marital status, and income were selected as demographic covariates a priori drawing from previous studies of snacking using NHANES data [41, 42]. Predictive marginal means, or probability weighted averages, are presented, with standard errors (SE). Pairwise comparisons of mean intake from snacks for each category by age group were performed using the Bonferroni-test at the 5% level of significance to adjust for multiple comparisons. Additionally, predictive marginal means are presented for each NHANES survey cycle to examine secular trends in daily snacking energy by contextual factor over the 2007–2018 survey periods.

## Results

A total of 19,252 children aged 1–19 y participated in the combined 2007–2018 cycles of NHANES; 2,169 cases from participants who did not report snacking occasions, or only reported snack occasions with 0 kcal were removed from the main analysis given the interest in examining the percentage of daily energy from snacking occasions. Additionally, participants having a diabetes diagnosis (n = 63), who were still being breast-fed (n = 121), and who were missing covariate data (poverty income ratio, n = 1,282; HH education, n = 418; HH marital status, n = 533) were excluded. The final analytical sample consisted of 14,666 children.

Participants were on average 9.6 (0.1) y, 49% female, 57% non-Hispanic White, 13% non-Hispanic Black, 22% Hispanic, and 9% other race and ethnicity (Table 1). Toddlers made up 11% of the sample, preschoolers 16%, school-age children 34%, and adolescents 39%. Among the HH of participating children, 50% were < 40 y, 29% had a college degree or higher, 75% were partnered, and 32% had a PIR < 125%.

**Table 1:** Socio-demographic characteristics of 14,666 U.S. children 1–19 y reporting snacking intake and participating in NHANES 2007-2018^a,b,c^

Child	n (%)
Age, y (mean(SE)^d^)	9.6 (0.1)
Gender	
Male	7447 (51)
Female	7219 (49)
Age group	
1 to 2	2240 (11)
3 to 5	2452 (16)
6 to 11	5089 (34)
12 to 19	4885 (39)
Race and ethnicity	
Non-Hispanic White	4664 (57)
Non-Hispanic Black	3340 (13)
Hispanic	4781 (22)
Other^e^	1881 (9)
**Head of household**	**n (%)**
Age	
< 40 y	8058 (50)
≥ 40 y	6608 (50)
Education	
Less than high school	3637 (18)
High school/some college	7783 (53)
College graduate or above	3246 (29)
Marital status	
Partnered	10,361 (75)
Not partnered	4305 (25)
Poverty income ratio^f^	
< 125%	6161 (32)
≥ 125%	8505 (68)

^a^Data were derived from DEMO files, NHANES 2007–2018, weighted.

^b^Snacking = eating occasions self-identified as snacks or other eating between meals (beverages and extended consumption) > 0 kcal

^c^NHANES = National Health and Nutrition Examination Survey

^d^SE = standard error

^e^Includes non-Hispanic Asian and other race, including multi-racial

^f^Calculated by dividing family income by the poverty guidelines specific to the survey year

### Overall differences across age groups

**Fig. 1:**
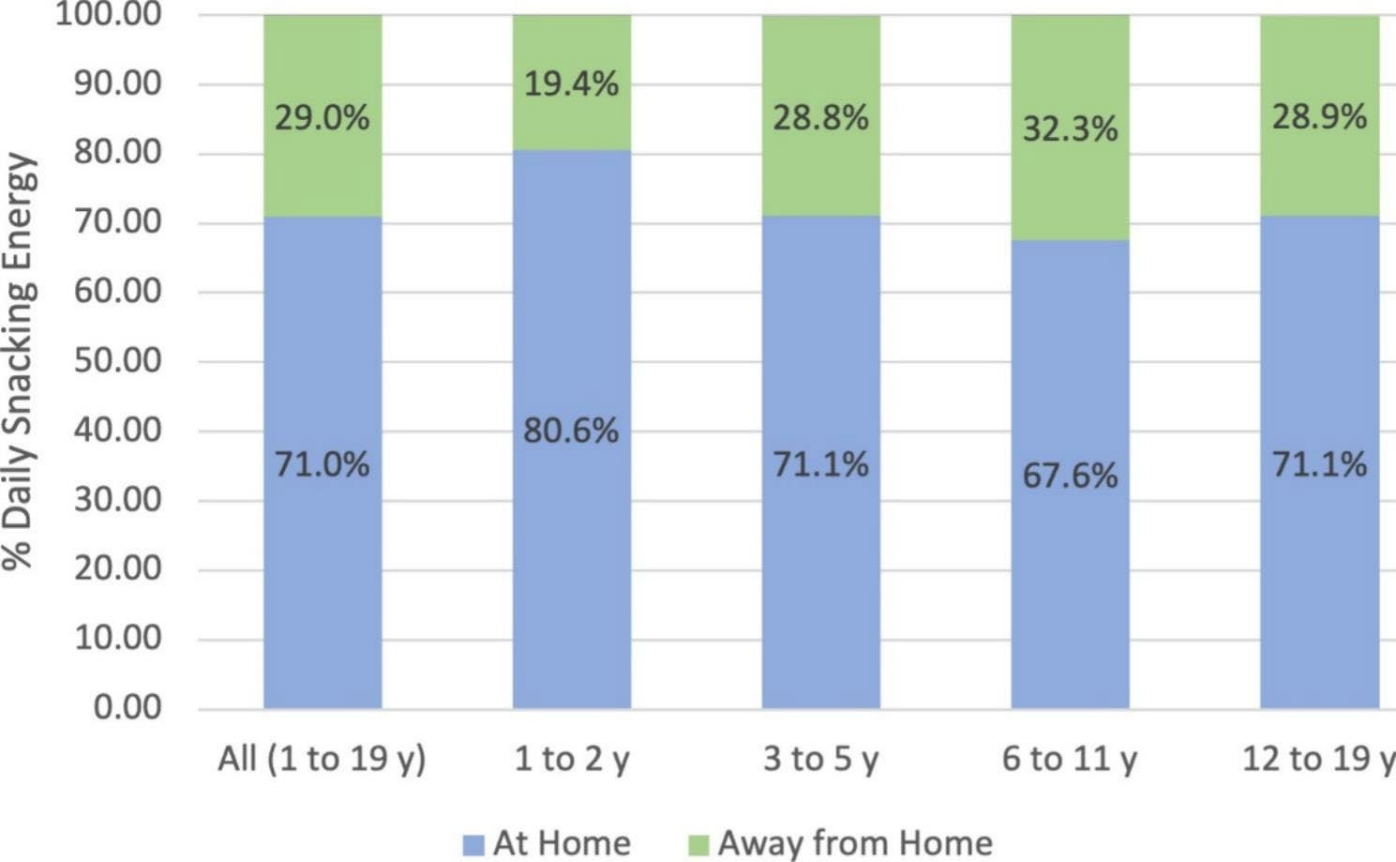
Percent daily snack energy from eating location by age group (n = 14,666). ^a^Eating locations identified as “unknown” are not represented in the graph (0.6% of the sample identified an eating location as “unknown”)

**Fig. 2:**
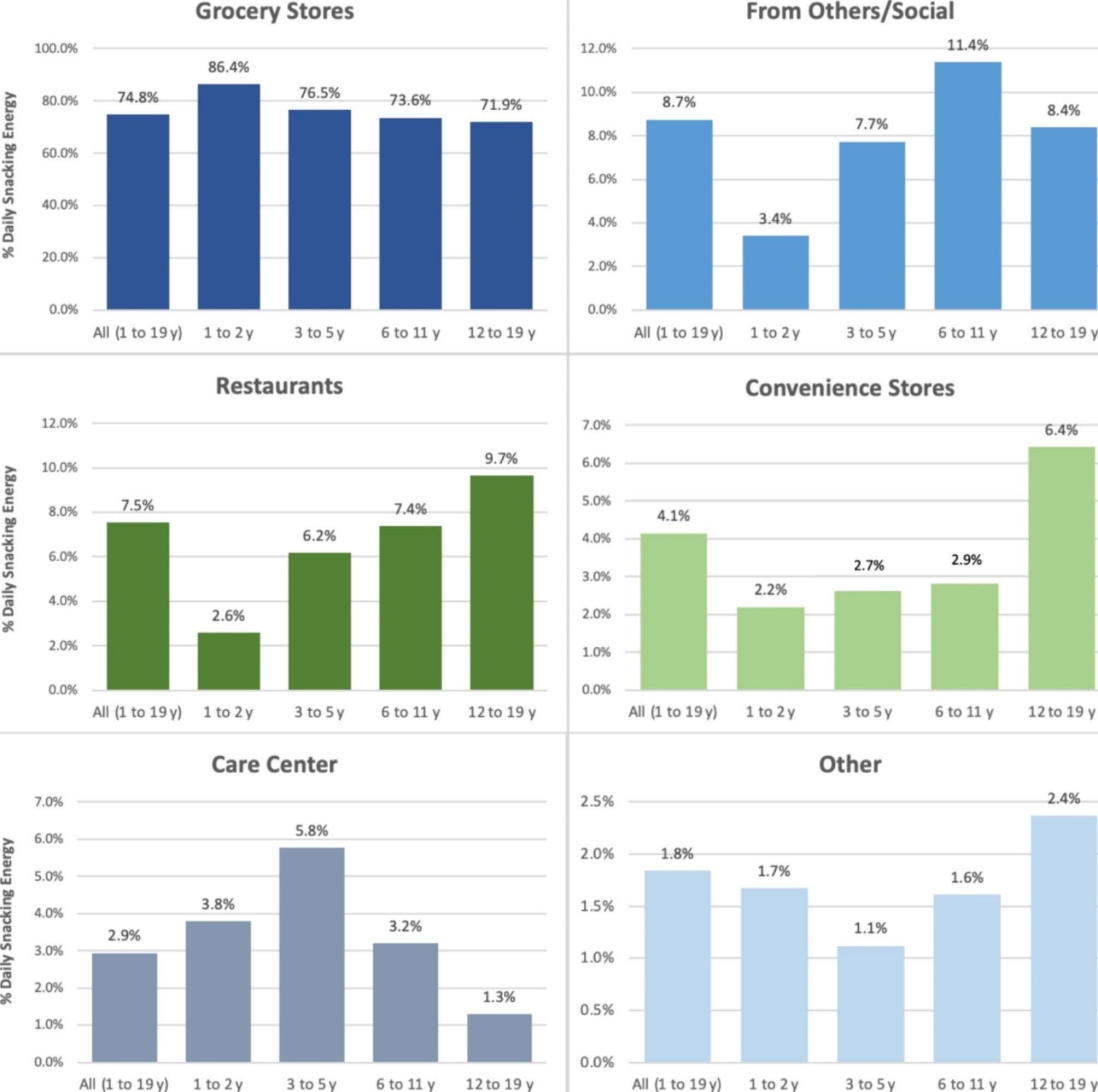
Percent Daily Snack Energy by Food Source Location and Age Group (n = 14,666)

**Fig. 3:**
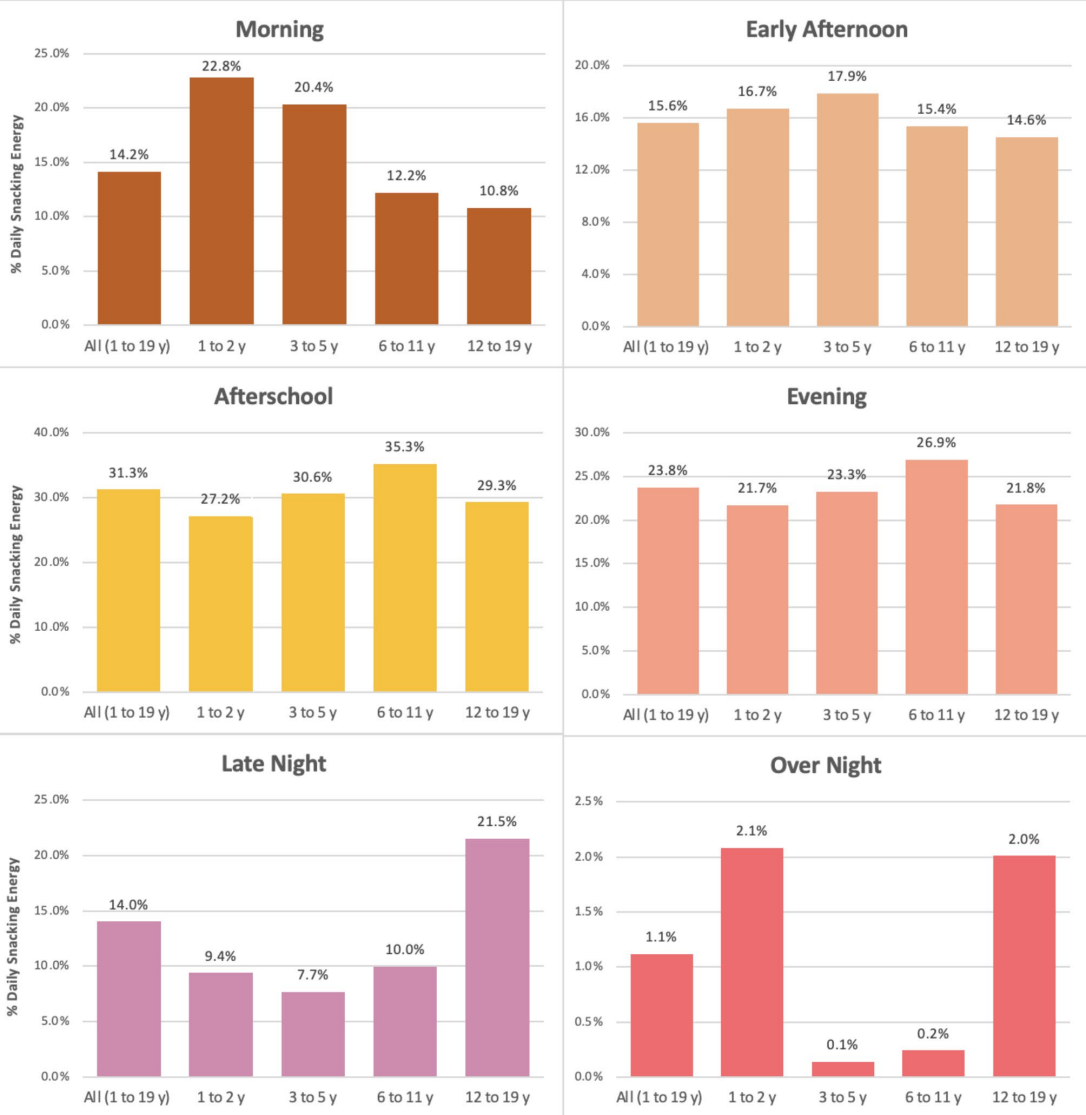
Percent Daily Snack Energy by Time of Day and Age Group (n = 14,666)

Toddlers 1–2 y consumed 431 kcal daily from snacks representing 31.7% of their daily energy intake, children 3–5 y consumed 475 kcal daily from snacks representing 29.1% of their daily energy intake, children 6–11 y consumed 519 kcal daily from snacks representing 26.1% of their daily energy intake, and children 12–19 y consumed 603 kcal daily from snacks representing 27.6% of their daily energy intake. Across all age groups, children 1–19 y consumed 71.0(0.5)% of daily snacking energy at home (Fig. 1). Children consumed the largest proportion of daily snack energy from foods/beverages obtained at grocery stores (74.8(0.5)%), followed by social sources (8.7(0.3)%), restaurants (7.5(0.3)%), convenience stores (4.1(0.2)%), school/childcare (2.9(0.2)%), and other (1.8(0.1)%) locations (Fig. 2). Children consumed the largest proportion of daily snack energy (31.3(0.6)%) in the afternoon/afterschool period (3pm-6pm), followed by evening (6pm-9pm; 23.8(0.5)%), early afternoon (12pm-3pm; 15.6(0.3)%), morning (6am-12pm; 14.2(0.3)%), and late night (9pm-12am; 14.0(0.5)%. Little snack intake occurred overnight (12am-6am; 1.1(0.1)%) (Fig. 3).

### Age-specific differences

Between group age-related differences are detailed in Supplementary Table 2. Below, key differences for each age group are summarized.

#### Toddlers (1–2 y)

Toddlers consumed the highest percentage of daily snack energy at home (80.6(0.9)%, all p < 0.001), and from foods/beverages obtained from grocery stores compared to all other age groups (86.4(0.9)%, all p < 0.001). Toddlers also consumed a greater percentage of daily snack energy during the morning (22.8(0.7)%) compared to school-age children and adolescents who did not consume appreciable snacking energy during this time (6–11 y: 12.2(0.6)%; 12-19y: 10.8(0.6)%, both p < 0.001).

#### Preschool-age children (3–5 y)

Preschool age children consumed a greater percentage of daily snack energy from school/childcare (5.8(0.5)%) compared to other age groups (p < 0.05 to < 0.001) and consumed a greater percentage of daily snack energy in the early afternoon (12pm-3pm; 17.9(0.8)%) compared to school-age children and adolescents (p < 0.05 to < 0.01).

#### School-age children (6–11 y)

Schoolage children consumed a greater percentage of daily snack energy from foods and beverages obtained from social sources (11.4(0.6)%) than any other age group (all p < 0.001). This group also consumed the greatest percentage of daily snack energy during the late afternoon/afterschool period (3pm-6pm; 35.3(0.9)%) and in the evening (6pm-9pm; 26.9(0.8)%) compared to all other age groups (p < 0.05 to < 0.001).

#### Adolescents (12–19 y)

Adolescents consumed a greater percentage of daily snack energy obtained from restaurants (9.7(0.5)%) and convenience stores (6.4(0.5)%) than other age groups (p < 0.001). This group also consumed the greatest percentage of daily snack energy in the late-night period compared to all other age groups (21.5(0.8)%, all p < 0.001).

### NHANES survey cycle differences

Interestingly there has been a steady increase in the percent of snacking energy consumed at home (p < 0.05) relative to a decrease in the percent of snacking energy consumed away from home (p < 0.05) since the 2007–2008 cycle (Supplementary Table 3). However, conversely, the percentage of snacking energy obtained from grocery stores has steadily decreased since 2007 (81–71%, p < 0.001) and the amount of snacking energy obtained from convenience stores (0–6.7%, p < 0.001) and restaurants (6.2–7.8%, p < 0.01) has increased. Overall, there were no differences in timing of snack intake across survey cycles.

## Discussion

The present analysis provides nationally representative evidence of developmental differences in the social and contextual factors influencing snacking behavior among U.S. children. Overall, U.S. children 1–19 y consumed the greatest percentage of total daily snack energy at home, from foods and beverages obtained from grocery stores, and in the late afternoon/afterschool period. While late afternoon/afterschool seems to be a key time for snacking across all age groups, other important age-specific differences in the timing of snacking intake emerged. Toddlers and preschool-age children consumed the greatest snack energy in the morning compared to other age groups, while school-age children had greater snack energy intakes in the late afternoon and evening, and adolescents had the greatest snack energy intake during the late-night period. Additionally, toddlers and preschool-age children consumed a greater percentage of daily snacking energy at home and from foods and beverages obtained from school/childcare compared to school-age children and adolescents. Among older children, key contexts for snacking included eating snacks away from home, and obtaining snacks socially, and from restaurants and convenience stores. These observations provide empirical evidence for developmental shifts in the timing and autonomy of snacking behavior and provide critical insights to inform future research.

Consistent with previous work, the findings of this analysis suggest that snacking among U.S. children primarily involves foods/beverages obtained at grocery stores and consumed at home [18, 43]. The proportion of daily snack energy from these sources decreased across age groups but were nevertheless the highest contributors to overall snack intake. These findings are consistent with 2003–2006 NHANES data in which roughly 63% of daily energy came from stores among children 6–11 y and adolescents aged 12–19 y [18]. Our findings also align with a 2009–2016 analysis of NHANES data that revealed that adolescents consume more added sugar, saturated fat, and sodium from food and beverage snacks consumed at home than those consumed away from home [16]. Collectively, these findings highlight the importance of family-level factors on snacking intake. Parents and caregivers act as primary agents of eating socialization among children by influencing the foods and beverages available in the home as well as through modeling eating behaviors, and using feeding styles and practices to guide children’s eating [44, 45]. The present findings highlight the need to articulate factors within the home that shape the quality and quantity of children’s snacking intake. Findings also point to the need to explicitly address snacking behaviors in family-based prevention efforts.

While children consumed most snacking energy at home, significant developmental differences emerged in major food source locations for snacking (e.g., where snacks were obtained). Schools and childcare centers were more prominent in early childhood (3–5 y), highlighting the potential role of early childhood education (ECE) food environments and policy impacts on snacking. The CACFP program, for instance, subsidizes snacking in childcare and afterschool programs that serve populations with low-income by providing reimbursements for snacks that follow nutrition program requirements [46]. Alternatively, restaurants and convenience stores were more important food source locations for snacking among adolescents than other age groups and interestingly, social sources (e.g., snacks obtained from others or as a “gift”) were important for school-age children (6–11 y). These findings are consistent with the observation that influences like peers and the broader food environment have an impact on eating outside the home among older age groups, and these factors should be included in recommendations and intervention efforts for these age groups [47]. For example, approaches that target peers and social networks might be impactful for older children, while policy, systems, and environmental (PSE) interventions that promote healthier food choices like soda taxes or added sugar warning labels, might have a positive impact on snacking selection and intake in adolescents [48–50].

The finding that snacking among toddlers occurred in the morning as well as the afternoon aligns with typical meal and snack patterns in childcare and early childhood settings. These patterns also reflect the transition from frequent milk feeding in infancy to a modified adult eating pattern during toddlerhood involving small meals and snacks [10]. That young children snack in the morning as well as afternoon may also reflect the commonly held notion that young children need snacks to meet nutritional needs and snacks act as a ‘hold over’ in between meals [44, 51]. In contrast, snacking among older children was concentrated in the afterschool period and evening periods and pronounced in the late-night period among adolescents. These findings are aligned with prior work and may reflect the greater autonomy older children have around snacking choices including greater ability to verbally articulate food wants/needs, to physically obtain and prepare foods/beverages, more time in the day spent eating without adult supervision (e.g., school), and greater purchasing power for obtaining foods and beverages outside of meals [13, 21].

This work contributes novel information to the understanding of contextual influences on snacking in pediatric populations. However, contributions of this work should be noted in the context of limitations. While NHANES employs rigorous methods for collecting dietary recalls, provides comprehensive U.S. population-representative data, and 24-hour dietary recalls remain the gold standard for dietary assessment in epidemiological studies, self-reported recalls are still subject to random and systematic measurement error including reporting/recall bias [29]. Next, while our findings were aligned with trends observed by eating location and timing of snack intake in a Canadian sample of children, this work is limited to a U.S. sample [43]. As snacking continues to increase as a prevalent eating behavior worldwide, trends should be examined in other populations. It should also be noted that bottled water and zero-calorie beverages are not represented in this analysis since they do not contribute to the percent of daily snack energy and were thus excluded. Although these items do not contribute energy to children’s diets, they may have implications for nutritional intake and growth that are not captured in this study. Also, given that a percent of daily snack energy was used as the outcome, those with a high percentage of daily snacking might be overrepresented in this analysis and future work should consider stratifying findings between those with high and low snack intake to determine if results are similar. Finally, there are many contextual factors that may influence snacking in children that were not captured due to limitations in the data available in NHANES. For example, concurrent behaviors like screen time are increasingly relevant and are understudied in relation to eating behavior, as well as other multi-level drivers of intake like family, peers, and food access [32, 44, 52]. The ability to comprehensively capture these influences remains limited by methodology (e.g., snacking definitions) and a need for more robust data collection approaches. For example, ecological momentary assessment or longitudinal cohorts could provide more in-depth insights on these factors and their impact on child diet and health.

## Conclusion

This work provides descriptive evidence of developmentally unique timing and contexts for snacking among U.S. children 1–19 y of age. This work highlights the need for future studies to provide robust evidence for age-specific recommendations around snacking that take contextual factors such as setting, food source location, and timing into consideration. This work can be used to inform public health efforts around snacking by providing information on the key contextual factors that influence where and when snacks are obtained and consumed by U.S. children.

## Electronic supplementary material

Below is the link to the electronic supplementary material.

Supplementary Material 1

Supplementary Material 2

Supplementary Material 3

## Data Availability

The NHANES datasets generated and analyzed in this research are publicly available at https://wwwn.cdc.gov/nchs/nhanes/search/DataPage.aspx?Component=Dietary.

## Abbreviations

U.S.: United States
NHANES: National Health and Nutrition Examination Survey
Y: years
CACFP: Child and Adult Care Food Program
SSB: Sugar-sweetened Beverages
Kcal: Energy/kilocalories
HH: Head of Household
PIR: Poverty-income ratio
SE: Standard Error
