# Supplementary material for: Age-related differences in eating location, food source location, and timing of snack intake among U.S. children 1–19 years
Source: Int J Behav Nutr Phys Act. 2023 Jul 26;20:90. doi: 10.1186/s12966-023-01489-z (PMC10369691; doi:10.1186/s12966-023-01489-z)
Supplement: Supplementary file 1 — Supplementary Material 1 [file 12966_2023_1489_MOESM1_ESM.docx]

**Supplementary File 1.** Snacking location and timing variable grouping and NHANES categories

| **Variable Categories** | **NHANES Code** | **NHANES Description** | **Percentage of daily snacking energy** |  |
| --- | --- | --- | --- | --- |
| *Source Location (DR1FS)* | | | | |
| **GROCERY STORES** | 1 | Store - grocery/supermarket | 75% |  |
|  | 28 | Store - no additional info | 0% |  |
| **CONVENIENCE STORES** | 27 | Store - convenience type | 4% |  |
| **CARE CENTER/SCHOOL** | 7 | Cafeteria in a K-12 school | 1% |  |
|  | 8 | Child/Adult care center | 1% |  |
|  | 9 | Child/Adult home care | 0% |  |
| **RESTAURANT** | 2 | Restaurant with waiter/waitress | 1% |  |
|  | 3 | Restaurant fast food/pizza | 5% |  |
|  | 4 | Bar/tavern/lounge | 0% |  |
|  | 5 | Restaurant no additional information | 0% |  |
|  | 6 | Cafeteria NOT in a K-12 school | 0% |  |
|  | 24 | Sport, recreation, or entertainment facility | 2% |  |
|  | 25 | Street vendor, vending truck | 1% |  |
| **SOCIAL** | 16 | From someone else/gift | 9% |  |
| **COMMUNITY/OTHER** | 10 | Soup kitchen/shelter/food pantry | 0% |  |
|  | 11 | Meals on Wheels | 0% |  |
|  | 12 | Community food program - other | 0% |  |
|  | 13 | Community program no additional information | #Dropped; only 14 people reported this category over 10 years and none reported in analytic sample |  |
|  | 14 | Vending machine | 0% |  |
|  | 15 | Common coffee pot or snack tray | 0% |  |
|  | 17 | Mail order purchase | 0% |  |
|  | 18 | Residential dining facility | 0% |  |
|  | 19 | Grown or caught by you or someone you know | 0% |  |
|  | 20 | Fish caught by you or someone you know | 0% |  |
|  | 26 | Fundraiser sales | 0% |  |
|  | 91 | Other, specify | 0% |  |
|  | 99 | Don't know | 0% |  |
|  | . | Missing | 0% |  |
| *Time of Day (DR1_020)* | | | | |
| **MORNING (6am-noon)** |  | >= "06:00:00" & < "12:00:00" |  |  |
| **EARLY NOON (noon-3pm)** |  | >= "12:00:00" & < "15:00:00" |  |  |
| **LATE NOON/AFTER SCHOOL (3-6pm)** |  | >= "15:00:00" & < "18:00:00" |  |  |
| **EVENING (6-9pm)** |  | >= "18:00:00" & < "21:00:00" |  |  |
| **LATE NIGHT (9pm-12)** |  | >= "21:00:00" & < "24:00:00" |  |  |
| **OVERNIGHT (12-6am)** |  | >= "00:00:00" & < "06:00:00" |  |  |
| *Intake Location (DR1_040Z)* | | | | |
| **AT HOME** | 1 | Yes |  |  |
| **NOT AT HOME** | 2 | No |  |  |
| **UNKNOWN** | 7 | Refused |  |  |
|  | 9 | Don't know |  |  |
|  | . | Missing |  |  |
