# Supplementary material for: Age-related differences in eating location, food source location, and timing of snack intake among U.S. children 1–19 years
Source: Int J Behav Nutr Phys Act. 2023 Jul 26;20:90. doi: 10.1186/s12966-023-01489-z (PMC10369691; doi:10.1186/s12966-023-01489-z)
Supplement: Supplementary file 2 — Supplementary Material 2 [file 12966_2023_1489_MOESM2_ESM.docx]

**Supplementary File 2.** Mean (SE) between age group differences in percentage of daily snack energy by eating location, food source location, and time of day (n=14,666)

| **Food Source Location** | | | | |
| --- | --- | --- | --- | --- |
| **Age (y)** | **% Daily Snack Energy** | **1 to 2** | **3 to 5** | **6 to 11** |
| *Grocery Stores* | | **M(SE)** | **M(SE)** | **M(SE)** |
| **1 to 2** | 86.4 |  |  |  |
| **3 to 5** | 76.5 | -9.8(1.3)*** |  |  |
| **6 to 11** | 73.6 | -12.8(1.1)*** | -3.0(1.2). |  |
| **12 to 19** | 71.9 | -14.4(1.4)*** | -4.6(1.4)** | -1.6(1.1) |
| *Convenience Stores* | | | | |
| **1 to 2** | 2.2 |  |  |  |
| **3 to 5** | 2.7 | 0.5(0.4) |  |  |
| **6 to 11** | 2.9 | 0.7(0.4) | 0.2(0.4) |  |
| **12 to 19** | 6.4 | 4.2(0.7)*** | 3.7(0.6)*** | 3.5(0.6)*** |
| *Care Center/School* | | | | |
| **1 to 2** | 3.8 |  |  |  |
| **3 to 5** | 5.8 | 2.0(0.7)* |  |  |
| **6 to 11** | 3.2 | -0.6(0.6) | -2.6(0.6)*** |  |
| **12 to 19** | 1.3 | -2.5(0.6)*** | -4.5(0.6)*** | -1.9(0.4)*** |
| *Restaurant* | | | | |
| **1 to 2** | 2.6 |  |  |  |
| **3 to 5** | 6.2 | 3.6(0.8)*** |  |  |
| **6 to 11** | 7.4 | 4.8(0.6)*** | 1.2(0.8) |  |
| **12 to 19** | 9.7 | 7.1(0.6)*** | 3.5(0.9)*** | 2.3(0.6)*** |
| *Social/From Someone Else* | | | | |
| **1 to 2** | 3.4 |  |  |  |
| **3 to 5** | 7.7 | 4.3(0.5)*** |  |  |
| **6 to 11** | 11.4 | 8.0(0.6)*** | 3.7(0.7)*** |  |
| **12 to 19** | 8.4 | 5.0(0.7)*** | 0.7(0.7) | -3.0(0.7)*** |
| *Community/Other* | | | | |
| **1 to 2** | 1.7 |  |  |  |
| **3 to 5** | 1.1 | -0.6(0.3) |  |  |
| **6 to 11** | 1.6 | -0.1(0.4) | 0.5(0.3) |  |
| **12 to 19** | 2.4 | 0.7(0.4) | 1.3(0.4)** | 0.8(0.4) |
|  | | | | |
| **Time of Day** | | | | |
| **Age (y)** | **% Daily Snack Energy** | **1 to 2** | **3 to 5** | **6 to 11** |
| *Morning (6am-noon)* | | **M(SE)** | **M(SE)** | **M(SE)** |
| **1 to 2** | 22.8 |  |  |  |
| **3 to 5** | 20.4 | -2.5(1.2) |  |  |
| **6 to 11** | 12.2 | -10.6(0.9)*** | -8.2(0.9)*** |  |
| **12 to 19** | 10.8 | -12.0(0.9)*** | -9.5(1.1)*** | -1.4(0.8) |
| *Early afternoon (noon-3pm)* | | | | |
| **1 to 2** | 16.7 |  |  |  |
| **3 to 5** | 17.9 | 1.2(1.0) |  |  |
| **6 to 11** | 15.4 | -1.3(1.0) | -2.5(0.9)* |  |
| **12 to 19** | 14.6 | -2.2(1.0) | -3.3(1.1)** | -0.8(0.8) |
| *Late afternoon/After School (3pm-6pm)* | | | | |
| **1 to 2** | 27.2 |  |  |  |
| **3 to 5** | 30.6 | 3.4(1.3)* |  |  |
| **6 to 11** | 35.3 | 8.0(1.4)*** | 4.6(1.2)*** |  |
| **12 to 19** | 29.3 | 2.1(1.3) | -1.3(1.3) | -5.9(1.3)*** |
| *Evening (6pm-9pm)* | | | | |
| **1 to 2** | 21.7 |  |  |  |
| **3 to 5** | 23.3 | 1.6(1.1) |  |  |
| **6 to 11** | 26.9 | 5.2(1.1)*** | 3.7(1.2)* |  |
| **12 to 19** | 21.8 | 0.1(1.3) | -1.5(1.3) | -5.2(1.2)*** |
| *Late Night (9pm-12am)* | | | | |
| **1 to 2** | 9.4 |  |  |  |
| **3 to 5** | 7.7 | -1.7(0.6) * |  |  |
| **6 to 11** | 10.0 | 0.6(0.7) | 2.3(0.8)* |  |
| **12 to 19** | 21.5 | 12.1(1.0)*** | 13.8(1.0)*** | 11.5(0.8)*** |
| *Over Night (12am-6am)* | | | | |
| **1 to 2** | 2.1 |  |  |  |
| **3 to 5** | 0.1 | -1.9(0.2)*** |  |  |
| **6 to 11** | 0.2 | -1.8(0.3)*** | 0.1(0.1) |  |
| **12 to 19** | 2.0 | -0.1(0.4) | 1.9(0.3)*** | 1.8(0.3)*** |
|  | | | | |
| **Eating Location** | | | | |
| **Age (y)** | **% Daily Snack Energy** | **1 to 2** | **3 to 5** | **6 to 11** |
| *At Home* | | **M(SE)** | **M(SE)** | **M(SE)** |
| **1 to 2** | 80.6 |  |  |  |
| **3 to 5** | 71.1 | -9.5(1.3)*** |  |  |
| **6 to 11** | 67.6 | -12.9(1.2) *** | -3.4(1.4) . |  |
| **12 to 19** | 71.1 | -9.5(1.3) *** | 0.01(1.5) | 3.4(1.4) . |
| *Away from Home* | | | | |
| **1 to 2** | 19.4 |  |  |  |
| **3 to 5** | 28.8 | 9.4(1.3)*** |  |  |
| **6 to 11** | 32.3 | 12.9(1.2)*** | 3.5(1.4). |  |
| **12 to 19** | 28.9 | 9.5(1.3)*** | 0.1(1.4) | -3.4(1.4). |
| *Unknown* | | | | |
| **1 to 2** | 0.0 |  |  |  |
| **3 to 5** | 0.2 | 0.1(0.1) |  |  |
| **6 to 11** | 0.03 | 0.02(0.03) | -0.1(0.1) |  |
| **12 to 19** | -0.01 | -0.02(0.03) | -0.2(0.1) | -0.04(0.03) |
