# Supplementary material for: Age-related differences in eating location, food source location, and timing of snack intake among U.S. children 1–19 years
Source: Int J Behav Nutr Phys Act. 2023 Jul 26;20:90. doi: 10.1186/s12966-023-01489-z (PMC10369691; doi:10.1186/s12966-023-01489-z)
Supplement: Supplementary file 3 — Supplementary Material 3 [file 12966_2023_1489_MOESM3_ESM.docx]

**Supplementary Table 3**. Mean (SE) of the percentage of daily snacking energy by food source location, time of day, and eating location, among U.S. children 1-19 years (n=14,666), by NHANES Survey Cycle.

| **Food Source Location** | | | | | | | | | | | | |
| --- | --- | --- | --- | --- | --- | --- | --- | --- | --- | --- | --- | --- |
|  | **STORE** | | **STORE - CONV.** | | **CARE CENTER/**  **SCHOOL** | | **RESTAURANT** | | **SOCIAL** | | **COMMUNITY/**  **OTHER** | |
| **NHANES Cycle** | *Mean* | *SE* | *Mean* | *SE* | *Mean* | *SE* | *Mean* | *SE* | *Mean* | *SE* | *Mean* | *SE* |
| **2007-2008** | 81.0 | 0.8 | -0.1 | 0.2 | 2.6 | 0.4 | 6.2 | 0.6 | 8.4 | 0.6 | 2.0 | 0.3 |
| **2009-2010** | 80.9 | 0.9 | 0.0 | 0.1 | 3.0 | 0.4 | 6.5 | 0.7 | 8.1 | 0.6 | 1.6 | 0.3 |
| **2011-2012** | 73.2 | 1.0 | 4.8 | 0.5 | 2.9 | 0.4 | 7.1 | 0.8 | 10.3 | 0.8 | 1.8 | 0.4 |
| **2013-2014** | 71.6 | 1.3 | 7.3 | 0.9 | 2.7 | 0.4 | 9.5 | 0.7 | 7.4 | 0.7 | 1.5 | 0.3 |
| **2015-2016** | 70.9 | 1.8 | 6.4 | 0.7 | 3.2 | 0.6 | 8.2 | 0.6 | 9.0 | 1.2 | 2.4 | 0.3 |
| **2017-2018** | 71.1 | 1.0 | 6.7 | 0.8 | 3.3 | 0.8 | 7.8 | 0.6 | 9.3 | 0.9 | 1.9 | 0.5 |
| ANOVA/ Wald Test | **p<.001** |  | **p<.001** |  | p= 0.95 |  | **p<.05** |  | p= 0.13 |  | p= 0.29 |  |

| **Time of Day** | | | | | | | | | | | | |
| --- | --- | --- | --- | --- | --- | --- | --- | --- | --- | --- | --- | --- |
|  | **MORNING**  **(6am-noon)** | | **EARLY AFTERNOON**  **(12pm-3pm)** | | **LATE AFTERNOON/**  **AFTER SCHOOL**  **(3-6pm)** | | **EVENING (6-9pm)** | | **LATE NIGHT**  **(9pm-12)** | | **OVER NIGHT**  **(12-6am)** | |
| **NHANES Cycle** | *Mean* | *SE* | *Mean* | *SE* | *Mean* | *SE* | *Mean* | *SE* | *Mean* | *SE* | *Mean* | *SE* |
| **2007-2008** | 13.5 | 0.7 | 15.6 | 0.9 | 31.0 | 1.3 | 24.3 | 1.1 | 14.6 | 1.3 | 1.0 | 0.4 |
| **2009-2010** | 14.1 | 0.6 | 16.3 | 0.7 | 30.9 | 1.3 | 23.4 | 1.3 | 14.2 | 0.6 | 1.0 | 0.2 |
| **2011-2012** | 15.2 | 1.1 | 14.7 | 0.5 | 31.6 | 1.0 | 22.6 | 1.0 | 14.6 | 1.2 | 1.3 | 0.4 |
| **2013-2014** | 14.0 | 0.8 | 15.4 | 0.9 | 32.6 | 1.2 | 22.5 | 1.1 | 14.2 | 0.6 | 1.4 | 0.3 |
| **2015-2016** | 14.7 | 0.8 | 15.0 | 0.8 | 32.2 | 1.4 | 24.0 | 0.8 | 13.1 | 1.0 | 1.0 | 0.2 |
| **2017-2018** | 13.5 | 0.6 | 16.8 | 0.9 | 29.4 | 2.0 | 26.0 | 1.3 | 13.4 | 1.4 | 1.0 | 0.2 |
| ANOVA/ Wald Test | p= 0.63 |  | p= 0.29 |  | p= 0.78 |  | p= 0.28 |  | p= 0.89 |  | p= 0.70 |  |

| **Eating Location** | | | | |
| --- | --- | --- | --- | --- |
|  | **AT HOME** | | **NOT AT HOME** | |
| **NHANES Cycle** | *Mean* | *SE* | *Mean* | *SE* |
| **2007-2008** | 69.4 | 1.0 | 30.7 | 1.0 |
| **2009-2010** | 68.3 | 1.3 | 31.6 | 1.3 |
| **2011-2012** | 71.8 | 1.1 | 28.2 | 1.1 |
| **2013-2014** | 71.3 | 1.4 | 28.7 | 1.4 |
| **2015-2016** | 71.6 | 1.2 | 28.4 | 1.3 |
| **2017-2018** | 73.5 | 1.1 | 26.4 | 1.1 |
| ANOVA/ Wald Test | **p<.05** |  | **p<.05** |  |
